# Atipical kawasaki disease with coronary aneurysm in infant

**DOI:** 10.1186/1824-7288-37-19

**Published:** 2011-04-17

**Authors:** Icilio Dodi, Vera Raggi, Marta Verna, Bertrand Tchana, Daniela Vignali, Maria Antonietta Bandello, Silvia Lacava, Gian Carlo Izzi, Aldo Agnetti

**Affiliations:** 1 Pediatric Hematology and Oncology Unit, Department of Pediatrics, Parma University Hospital, Italy; 2 School of Specialization in Pediatrics, School of Medicine, University of Parma, Italy; 3 Pediatric Cardiology Unit, Department of Pediatrics, Parma University Hospital, Italy

## Abstract

Kawasaki disease is an acute febrile disease of unknown etiology, characterized by systemic vascular inflammation involving the small and medium sized arteries, with a predilection for the coronary arteries. It represents the leading cause of acquired heart diseases in children in developed countries. Diagnosis, difficult because of the clinical characteristics of the disease with typical signs and symptoms appearing sequentially and not simultaneously, may be even more complicated in case of unusual presentation, leading to delay in recognition, particularly in infant in whom a higher incidence of coronary arteries aneurysms has been reported. A high index of suspicion of Kawasaki disease must be maintained in case of prolonged fever in these patients. Timely appropriate treatment is essential to avoid severe sequels. We report the case of a 2 months old male infant with persistent febrile episode, transferred to us from another institution, who presented on echocardiography giant aneurysms on both coronary arteries.

## Introduction

Kawasaki disease is an acute febrile disease of unknown etiology, characterized by systemic vascular inflammation involving the small and medium sized arteries, with a predilection for the coronary arteries, first describe by Tomisaku Kawasaki in Japan in 1967 [1,2]. The disease, counts for the leading cause of acquired cardiac disease in the developed countries, with an annual estimated incidence of 112 cases/100000 children in Japan and 2.9 - 6.9 cases/100000 children in Europe. About 80% of the children with Kawasaki disease are younger than 5 years of age with the peak of incidence between 6 and 11 months. The disease is rare in neonatal and infant period (only 1.6% of patients are under 90 days of age) [3] and extremely rare in adolescents and adults. Hospitalization rate of children under 5 years of age in the US was about 20.8/100000 in 2006 and remained constant from 1997 to 2007 [4-6]. The diagnosis relies only on fever associated with transient typical signs and symptoms appearing sequentially, usually not simultaneously present at the time of physical examination, therefore requiring an extremely precise case history. Difficulty in the diagnosis is increased by unusual and incomplete presentation, that may lead to delay in recognition and treatment and therefore severe sequels particularly in infant who are reported to have a higher incidence of coronary aneurysms [7-13]. Prompt and appropriate treatment with intravenous immunoglobulin (IVIG) associated with acetyl salicylic acid significantly reduces the risk of cardiac complications [14-18]. Among the untreated patients 25% of patients develop cardiac complications, declining to 5% in the patients who underwent the appropriate treatment 16. We report on a 2 month old male infant with persistent fever, transferred to us from another institution, who presented giant aneurysms of both coronary arteries on echocardiography.

## Case Report

**Figure 1:**
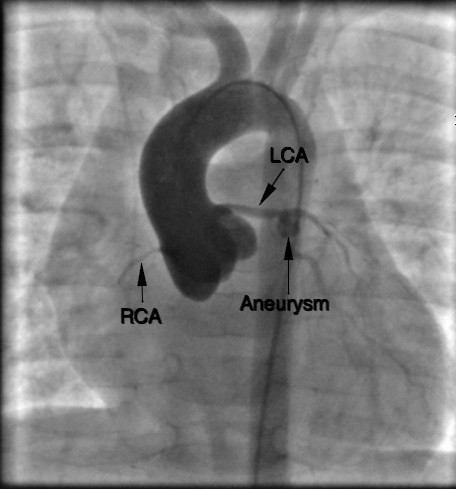
**LCA: main left coronary artery dilated with normal distribution and perfusion beyond the aneurysm**. Aneurysm: aneurysm of 5 mm in diameter at the bifurcation of the left coronary artery. RCA: thin right coronary artery, interrupted at 18 mm from its origin.

A 2 month old male infant was referred to our Hospital by another institution where he was admitted one week earlier for a two day history of fever, loss of appetite and rash. At that time laboratory studies showed an elevation of white blood cells (WBC 19.290/mm3). Chest x-ray and abdominal, cerebral and cardiac ultrasound were performed and urine, blood and cerebrospinal fluid (CSF) samples for culture were obtained. All these tests were negative. Therefore a systemic antibiotic therapy was started with ampicillin and gentamicin, and after two days switched to ceftriaxone and teicoplanin. Moreover a low dose of IGIV was given. On arrival in our Institution the patient was still febrile. Physical examination showed skin pallor, conjunctivitis and flushed lips, tachycardia, with normal heart sounds, no murmur, tachypnea; an echocardiography was performed, which resulted normal, as well as Chest X-ray, abdominal and cerebral ultrasound. Lumbar puncture was also performed and blood samples were obtained for complete blood cell count and culture. Laboratory studies showed increased WBC (28.830/mm3), platelets (575000/mm3) and Reactive Protein C (RCP 308 mg/L) and low hemoglobin (7.3 g/dL). The infant underwent red blood cell transfusion and antibiotic therapy with ampicillin and gentamicin was continued. On the second day ankle edema appeared while fever disappeared; blood examination revealed a significant reduction of RCP (72.4 mg/L) and WBC (21.910/mm3) with platelets persistently increasing (909000/mm3). The child remained well and apyretic for 7 days, then fever recurred, associated with irritability, tachycardia, skin rash on physical examination, and RCP at 239 mg/L (normal range 0 - 5 in our laboratory) and further increase of platelets (1170000/mm3) on laboratory investigations. The scheduled echocardiography was immediately performed and showed mitral valve insufficiency, pericardial effusion and severe anomalies on both coronary arteries: 3 aneurysms from 5 to 6 mm in diameter on the right coronary artery; left main coronary, normal proximally, was progressively dilated distally, as well as the circumflex artery, and there was a tubular aneurysm of 5 mm on the left anterior descending artery. Infusion of IVIG (2 gr/Kg in a single dose in twelve hours) and administration of acetyl salicylic acid (100 mg/Kg in 4 doses) was immediately started along with low molecular weight heparin (LMWH). Rapid improvement in clinical status, a significant decrease in RCP and platelet values (RCP 139 mg/L, platelets 882000/mmc) with complete normalization 20 days after IVIG infusion was observed. LMWH was then switched to Warfarin. The child was discharged 40 days after admission with acetyl salicylic acid and warfarin therapy, monitored by CoaguCheck System to maintain INR between 2.0 and 2.5. During follow up coronary aneurysms gradually improved. The last cardiac ultrasound showed a 5 mm left coronary artery aneurysm but right coronary artery was not observable. Mitral valve insufficiency and pericardial effusion were no longer present. A Cardiac catheterization was performed to study the coronary arteries which confirmed the presence of a 5 mm left coronary artery aneurysm with thrombus, and showed the interruption of the right coronary artery 18 mm from its origin (Figure 1). Currently the child receives acetyl salicylic acid 5 mg/Kg and warfarin 0.28 mg/Kg.

## Discussion

Diagnosis of Kawasaki's disease is a demanding challenge because it relies essentially on clinical criteria, with typical signs and symptoms similar to those present in different common children's diseases, appearing sequentially and fleeting. In our case an a posteriori audit of the first week report showed that the patient presented a fleeting episode of palm erythema along with edema of the hands and feet. Erroneous treatment due to mistake in the interpretation of signs and symptoms may alter clinical status and feature without modifying the course of the disease leading to a delay in the diagnosis and the timing of proper therapy. In our case the patient was given a low dose of IVIG which cancelled the fever and improved the clinical status for a week which only transiently slowed down the inflammatory process, leading to significant delay in the diagnosis and severe sequels. The key is a precise and well-done case history, and a close physical examination. Laboratory studies, particularly inflammatory indexes such as RCP may be helpful in the presence of clinical signs. Abnormal platelet count along with clinical signs and RCP value is revealing but is a late index which usually appears on the 12th - 14th day of disease whereas treatment should be given between the 6th and the 8th day. In our case abnormal platelet count along with a revision of the history and the other laboratory data should have raised the suspect of Kawasaki disease even in the presence of a completely negative echocardiography with the rapid disappearance of the fever. Unusual presentation is an important risk factor for delay in the diagnosis and severe cardiac sequels. Infants younger than one year of age are at the highest risk for coronary aneurysms and frequently show atypical forms of the disease [19,20]. In this category of patients, in case of fever lasting more than three consecutive days, Kawasaki disease should be considered in differential diagnosis.

Delayed diagnosis, along with incomplete clinical manifestation, has been suggested to be the major contributor to the development of coronary artery aneurysms. Timely appropriate treatment is essential for the avoidance of serious sequels in Kawasaki disease, particularly coronary aneurysms, especially in infants younger than one year of age in whom a high index of suspicion of Kawasaki disease must be maintained in case of prolonged fever. The key relies on a precise, detailed and complete case history and careful clinical evaluation and monitoring.

## Competing interests

The authors declare that they have no competing interests.

## Authors' contributions

ID: prepared the manuscript and search the literature

VR: Collected the data, participate in the search of literature

MV: participate in the collection of the data

BT: made the diagnosis, made the echocardiography, review the manuscript

DV: participate in the collection of the data

MAB: made the diagnosis, participate in the preparation of the manuscript

SL: participated in the collection of the data

GI: review the manuscript

AA: made the cardiac catheterization

All authors read and approved the final manuscript.

## Consent

Written informed consent was obtained from the patient for publication of this case report and accompanying images. A copy of the written consent is available for review by the Editor-in-Chief of this journal.
